# Spatial Organization of Slit-Confined Melts of Ring Polymers with Nonconserved Topology: A Lattice Monte Carlo Study

**DOI:** 10.1021/acs.macromol.3c01320

**Published:** 2023-09-28

**Authors:** Mattia Alberto Ubertini, Angelo Rosa

**Affiliations:** Scuola Internazionale Superiore di Studi Avanzati (SISSA), Via Bonomea 265, 34136 Trieste, Italy

## Abstract

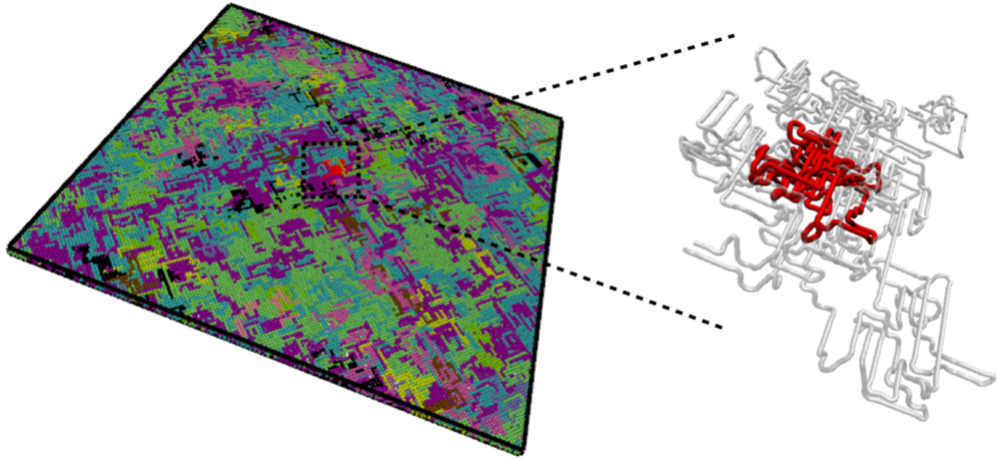

We present Monte Carlo computer simulations for melts of semiflexible randomly knotted and randomly concatenated ring polymers on the fcc lattice and in slit confinement. Through systematic variation of the slit width at fixed melt density, we explore the influence of confinement on single-chain conformations and interchain interactions. We demonstrate that confinement makes chains globally larger and more elongated while enhancing both contacts and knottedness propensities. As for multichain properties, we show that ring–ring contacts decrease with the confinement, yet neighboring rings overlap more as confinement grows. These aspects are accompanied by a marked decrease in the links formed between pairs of neighboring rings. In connection with the quantitative relation between links and entanglements in polymer melts recently established by us [UbertiniM. A.; RosaA.Macromolecules2023, 56, 3354–33623718124510.1021/acs.macromol.3c00278PMC10173697], we propose that confinement can be used to set polymer networks that act softer under mechanical stress and suggest a viable experimental setup to validate our results.

## 1 Introduction

Recent years have witnessed a growing interest in the design of so-called *smart* materials, such polycatenanes and polyrotaxanes,^1,2^ whose microscopic components are constituted by ring polymers interlocked to each other by topological links that can be artificially synthesized following precise chemical routes. Interestingly similar devices can be also prepared by employing biological components, mainly DNA plasmid rings^3^ which interlock with each other through the action of the enzyme *topoisomerase-II* and form a molecular state termed *Olympic* hydrogel which was first theorized by de Gennes in 1997.^4^ Remarkably, similar molecules can be also found in Nature: a classical example is the kinetoplast DNA^5^ present in the mitochondria of certain *Trypanosoma* parasites.

Similarly to covalent bonds stabilizing the shape of a molecule, topological links remain stable at room temperature, which guarantees the corresponding molecule to maintain a relatively well-characterized spatial conformation. On the other hand, since the single-ring constituents are not rigid objects but fluctuate^6^ as ordinary polymers typically do,^7,8^ these molecules display unusual mechanical properties under stress and tunable viscoelasticity that can be exploited in a wide number of practical applications (molecular machines and drug delivery,^9,10^ to name a few), thus justifying the adjective “smart” employed for these materials.

The preparation of topological materials with well-designed properties is a delicate balance between many parameters: indeed, several numerical studies^11−14^ have characterized the topological state of systems made up of randomly concatenated and knotted polymer rings, and have shown that the resulting networks can be controlled using experimentally tunable parameters such as the length of the polymer chain, the density of the polymer solution, and the bending stiffness of the polymer fiber. So far, though, *geometric confinement* as a way to drive the synthesis of concatenated ring networks has received considerably less attention. Yet, recent experiments^15^ performed on kinetoplast DNA^5^ at varying degrees of *slit confinement* have foreseen the possibility of exploiting geometric constraints to bias the synthesis of a DNA-based network, similarly to the one discussed in ref (3).

In this work, we explore how geometric constraints under the form of slit confinement can affect the structural properties of systems of strand-crossing rings. For this purpose, we perform extensive dynamical simulations of highly entangled systems of randomly concatenated and knotted rings employing the kinetic Monte Carlo algorithm introduced by us^13^ for studying these systems at bulk conditions. Varying the degree of confinement, we quantify its influence on the metric properties of the rings, which present interesting nonmonotonous behavior, as well as topological ones; in particular, knotting probability is highly enhanced by reducing the height of the slit, while the linking between the rings is diminished. These findings suggest that geometric confinement can be used as a powerful tool to control the topology of the resulting networks and their elastic properties.

This paper is structured as follows. In Section 1, we present and discuss the Monte Carlo lattice polymer model, introduce the notation, and explain how to detect and compute topological invariants for the characterization of knots and links in the system. In Section 2, we present the main results of our work, while in Section 3, we provide some discussion and conclusions regarding the role of slit confinement in shaping both single-chain and interchain properties of the resulting polymer networks. Additional figures are included in the Supporting Information (SI).

## 2 Model and Methods

### 2.1 Polymer Model

We consider polymer melts made of *M* randomly concatenated and randomly knotted ring polymers of *N* = 320 monomers each on the fcc lattice; the fcc unit step *a* is taken as our unit length. The simulations are based on the kinetic Monte Carlo (kMC) algorithm introduced by us in ref (13). Since then, the algorithm has been variously applied to study melts of nonconcatenated and unknotted rings^16^ and the connection between entanglements and physical links in semiflexible chain melts.^14^ In this article, we limit ourselves to summarizing the essential details of the numerical protocol, while referring the reader to our past works for more details.

Essentially, the polymer model takes into account: (i) chain connectivity, (ii) bending stiffness, (iii) excluded volume, and (iv) topological rearrangement of polymer chains. Finally, and for the first time, in this work, we consider (v) slit confinement in the model. For the implementation of chain dynamics, the following combination of MC moves—that automatically take into account excluded volume interactions—are used:

(a) Topology-*preserving* moves (termed *Rouse-like* and *reptation-like*, see ref (13)) that automatically enforce excluded volume interactions. By construction, these moves enable two (and no more than two) consecutive bonded monomers along each single chain to occupy the same lattice site: by allowing to store contour length along the polymer filament, this numerical “trick” makes the chains locally elastic and facilitates global chain equilibration. Because of that, the bond length is a fluctuating quantity with mean value = ⟨*b*⟩: in particular, the latter is insensitive to confinement (the measured values for ⟨*b*⟩ are reported in Table 1). In this way, the mean polymer contour length is *L* = *N*⟨*b*⟩ and, similarly, the mean contour length of a subchain of *n* monomers is .
(b) Topology-*changing* moves^13^ that induce random strand crossings between nearby polymer filaments at a tunable rate: we set this rate to 10^4^ kMC elementary steps, consistent with our previous works.^13,14,16^ Strand crossings between filaments of the same ring can result in the creation or destruction of knots, while inter-ring crossings may cause either catenation or decatenation. The model has been shown to exhibit dynamical behavior consistent with the experiments,^3^ specifically dynamic “fluidization” of the rings due to topological violations through strand crossings. Thus, by performing simulations of strand-crossing rings, we sample the ensemble of the network structures formed by randomly concatenated and knotted rings at the given density and in slit confinement (see below for details).

Then, bending stiffness is modeled in terms of the Hamiltonian (in Boltzmann units, κ_B_*T*)
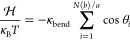
1where κ_bend_ = 2 is the bending stiffness and θ_*i*_ is the angle between consecutive bonds along the chain, with periodic conditions—due to ring geometry—assumed for all of the chains. By fixing the monomer number per fcc lattice site equal to ,^13,14,16^ the chosen bending stiffness corresponds to the chain Kuhn segment ,^16^ which is high enough to guarantee that distinct polymers are in an effective highly entangled state.

**Table 1:** Values of Physical Parameters for the Ring Polymer Melts Investigated in This Paper^a^

*H*/*a*	*Ĥ*	*M*	⟨*b*⟩/*a*
2.12	0.30	420	0.656
3.53	0.50	422	0.658
4.95	0.70	420	0.659
6.36	0.90	427	0.659
7.78	1.10	420	0.660
10.61	1.51	420	0.660
13.43	1.91	422	0.660
17.68	2.51	430	0.660
20.51	2.91	433	0.660
bulk	–	420	0.663

a *a* is the unit distance of the fcc lattice, and the monomer number per fcc lattice site is equal to , see text and refs (13,14,16) for details. (i) *H*, height of the slit. (ii) , ratio between the height of the slit and the root-mean-square gyration radius of rings in bulk (i.e., no confinement) conditions. (iii) *M*, total number of simulated chains in the melt. (iv) ⟨*b*⟩, mean bond length.^17^

**Figure 1:**
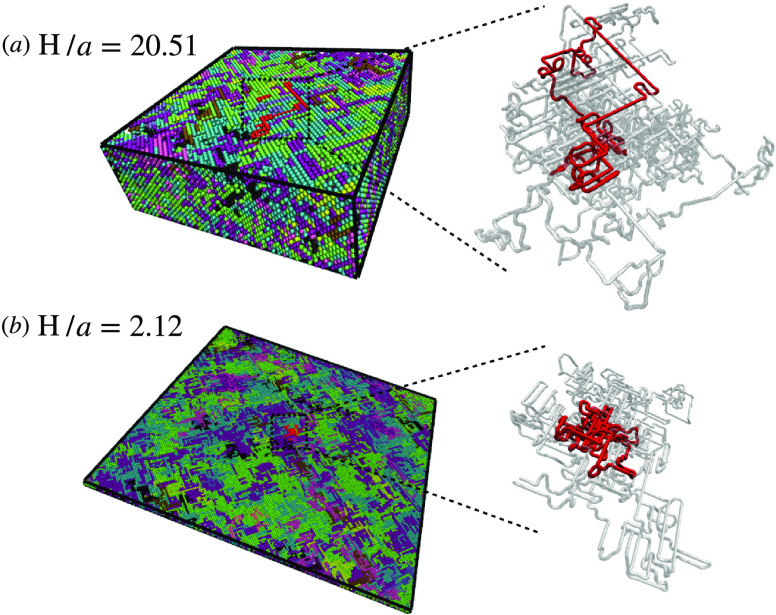
Ring melt conformations under slit confinement. The figure illustrates the two extreme cases of mild (*H*/*a* = 20.51, panel (a)) and tight (*H*/*a* = 2.12, panel (b)) confinement, where *H* is the thickness of the slit and *a* is the lattice unit (see Section 2.1 for details). On the left part of each panel, the full melt is shown with each ring in a different color to ease the visualization. On the corresponding right part, a zoom-in view of a typical ring conformation (in red) is presented alongside the neighboring rings (in faint gray) to which the red ring is linked (see Section 3.2.2).

Finally, the ring polymers are subject to slit confinement. This particular form of constraint is imposed by forcing the chains to move on the fcc lattice, with periodic boundary conditions on the *xy*-plane and hard boundaries in the *z*-direction placed in *z* = 0 and *z* = *H*. We vary the height of the box *H* to study different confinement regimes while adjusting the lateral box sides *L*_*x*_ = *L*_*y*_ to keep density constant. The degree of confinement is quantified by the ratio , expressing the ratio between the height, or width, of the slit *H* and the root-mean-square gyration radius (see definition 4), ,^16^ of rings in bulk conditions. We investigate the system’s behavior from highly confined (*Ĥ* ≃ 0:30) to mildly confined (*Ĥ* ≃ 2:91) regimes and systematically compare the results with the corresponding values in bulk. Wherever appropriate, we have also compared the systems here with melts of unknotted and nonconcatenated rings in bulk.^16^ We simulate *M* ≃ 420 chains, comprising a total of *N* × *M* ≃ 134,400 monomers, with *M* slightly adjusted to maintain a constant density (see Table 1 for specific numbers). Typical melt conformations (with corresponding zoomed-in views of a single ring and the neighbors to which it is linked) for the two situations of mild (*H*/*a* = 20.51) and tight (*H*/*a* = 2.12) confinement are shown in Figure 1 (panels (a) and (b), respectively).

To assess meaningful chain statistics and as in our other works^13,14^ on similar polymer systems, we run simulations long enough in order to get properly equilibrated melts. This is visualized in Figure S1 in the SI, which shows plots of the monomer time mean-square displacement in the frame of the center of mass of the corresponding chain (the so-called *g*_2_(18)) as a function of the MC simulation time τ_MC_. As known, provided long enough simulations are available, *g*_2_ displays a plateau that is indicative of the equilibration of the system. All our systems display corresponding plateaus, demonstrating that equilibration has been reached for all of the cases considered. Accordingly, the time scale to reach the corresponding plateau corresponds to the portion of the trajectory that has been discarded from the computation of the relative observables.

### 2.2 Detection of Knots and Links

In order to characterize the topological states of the rings in the melt, we follow closely the pipeline recently developed by us.^14^ Specifically, we employ a numerical algorithm that “shrinks” or simplifies each ring to its “primitive” shape, i.e., without violating topological constraints: in this way, we detect knots and links at any order, i.e., pairwise links as well as three-chain links like the *Borromean* ring configuration 6_2_^3^ (see Section 2.3 for knots and links notations). The algorithm is able to return the irreducible knotted or linked structure, which we further characterize by computing their topological invariants. For knots, in particular, we compute the corresponding Jones polynomial^19^ using the Python package *Topoly*.^20^ Instead, for two-body links we compute the Gauss linking number (GLN)
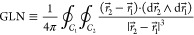
2which gives the number of times two closed loops  and  parametrized, respectively, by coordinates *r⃗*_1_ and *r⃗*_2_ wind around each other. While unconcatenated rings have GLN = 0, it is known that concatenated pairs exist with GLN = 0 (for instance, the so-called *Whitehead* link configuration 5_1_^2^). In these “pathological” cases, the ones detected via our shrinking algorithm were successively identified by computing the Jones polynomial using *Topoly* again. We compute the Jones polynomials also for three-chain irreducible links (for instance, Borromean rings) where a pairwise topological invariant such as the GLN fails (Section 3.2.2).

### 2.3 Notation

As for rings’ metric properties, for some observables  which can be expressed as a function of monomers’ coordinates, we study separately the contributions  and , respectively, perpendicular (or, transverse) and parallel to the plane of the slit (which, by construction (see Section 2.1), coincides with the *xy*-plane).

As for rings’ topological properties, in referring to a given knot or link we employ the conventional notation illustrated in the book by Rolfsen.^21^ Namely, a knot or a link is defined by the symbol *K*_*i*_^*p*^, where *K* represents the number of irreducible crossings of the knot (or the link), *p* is the number of rings that take part in the topological structure (e.g., *p* = 2 for two-chain links), and *i* is an enumerative index assigned to distinguish topologically *nonequivalent* structures having the same *K* and *p*.

## 3 Results

### 3.1 Single-Chain Properties

#### 3.1.1 Rings’ Size and Shape

First, we characterize the impact of slit confinement on the size and shape of the rings. To this purpose, for each ring of the system, we compute the 3 × 3 symmetric gyration tensor *Q*_*αβ*_ = *Q*_*βα*_(α, β = *x*, *y*, *z*) defined as
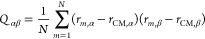
3where *r*_*m*,α_ is the α-th Cartesian component of the spatial position *r⃗*_*m*_ of monomer *m* and  is the center of mass of the chain. The mean eigenvalues of *Q* ordered in descending order, ⟨λ_1_^2^⟩ ≥ ⟨λ_2_^2^⟩ ≥ ⟨λ_3_^2^⟩, quantify the mean spatial elongations of the polymers on the corresponding principal axes, while the mean value of the trace of *Q*, ⟨tr*Q*⟩ = ∑_α = 1_^3^⟨λ_α_^2^⟩, is equal to the mean-square gyration radius or size
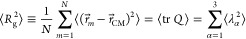
4of the chain.

**Figure 2:**
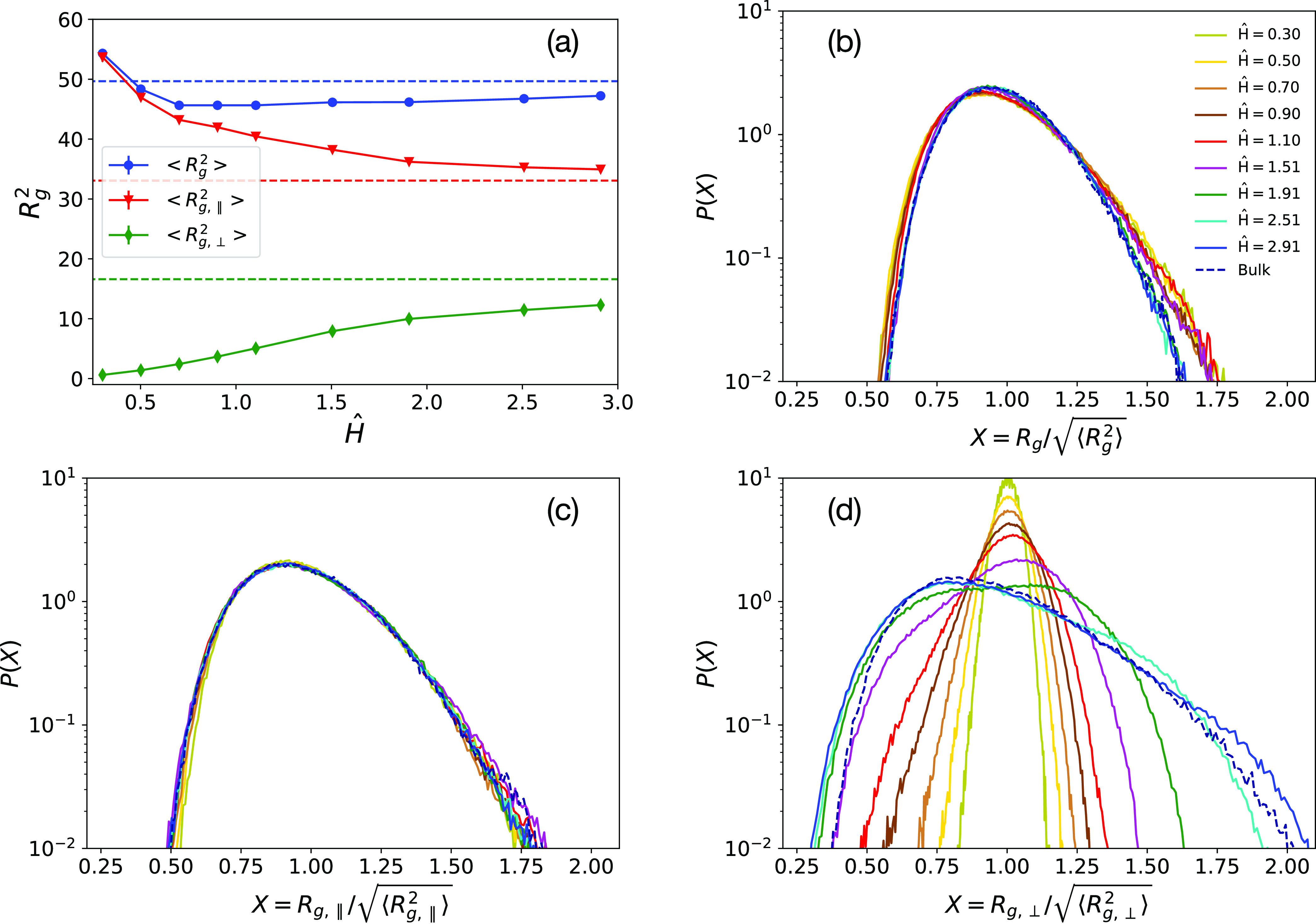
(a) Ring mean-square gyration radius (⟨*R*_g_^2^⟩) with its parallel (⟨*R*_g,∥_^2^⟩) and transverse (⟨*R*_g,⊥_^2^⟩) components as a function of the degree of confinement *Ĥ* (see Section 2.1 for details). The dashed lines are for the values of the bulk system (i.e., no confinement). Error bars are smaller than the symbol’s size. (b–d) Scaling plots for, respectively, distribution functions of the ring gyration radius  and of its parallel  and transverse  components, at different degrees of confinement *Ĥ* (see legend in panel (b)). The dashed line in each panel corresponds to the reference distributions under bulk conditions.

The results for ⟨*R*_g_^2^⟩ (eq 4) and the perpendicular and parallel components, ⟨*R*_g,⊥_^2^⟩ and ⟨*R*_g,∥_^2^⟩, are reported in Figure 2. As *H* decreases, the transverse component ⟨*R*_g,⊥_^2^⟩ decreases (green curve in Figure 2a) as expected. Conversely, the parallel component ⟨*R*_g,∥_^2^⟩ grows with confinement (red curve in Figure 2a) because the ring is forced to spread along the plane of the slit. Together, these two effects produce a characteristic nonmonotonic behavior in the overall ⟨*R*_g_^2^(*Ĥ*)⟩ (blue curve in Figure 2a) with the minimum attained around *Ĥ* ≃ 0.7, i.e., where confinement effects are expected to become more pronounced. Interestingly, for high confinement (≃0.3), the rings are markedly larger than the bulk reference (blue dotted curve in Figure 2a). In a previous study^22^ of randomly concatenated rings under slit confinement, the nonmonotonic behavior was also observed but the swelling compared to the bulk state was not seen. We attribute this discrepancy to the fact that, in the previous work, rings without excluded volume were considered, which could have favored more compact conformations.

Beyond average values, we have also computed the corresponding probability distributions, *P*(*R*_g_), *P*(*R*_g,⊥_), and *P*(*R*_g,∥_), and represented each of them (see Figure 2, panels (b) to (d)) in the corresponding scaled variable to ease comparison. While the distributions of the parallel component of the gyration radius are fundamentally unaffected by confinement (Figure 2c), those of the normal components (see Figure 2d) undergo a significant change in shape as the confinement becomes stronger, in particular becoming more peaked. Together these changes produce an interesting effect on the distributions of the full gyration radius (Figure 2b), which are characterized by higher tails for the systems under confinement. This suggests that under confinement rings assume more heterogeneous sizes.

**Figure 3:**
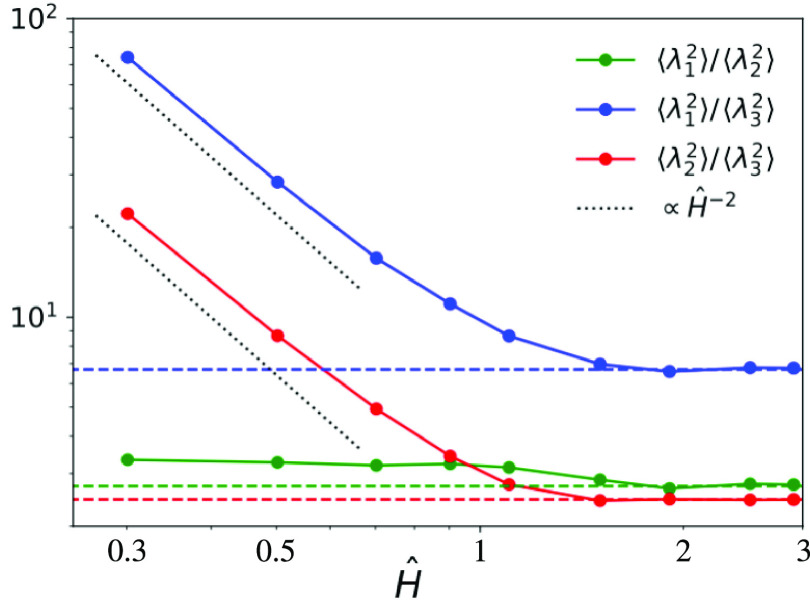
Ratios between the mean eigenvalues (⟨λ_1_^2^⟩, ⟨λ_2_^2^⟩, and ⟨λ_3_^2^⟩) of the ring gyration tensor *Q* (eq 3) as a function of the degree of confinement *Ĥ* (see Section 2.1 for definition). Dotted lines (∼*Ĥ*^–2^) describe the behavior under strong slit confinement, in agreement with the blob-like picture *à la* de Gennes (see Section 3.1.1 for details). Dashed horizontal lines correspond to the bulk reference values of the three ratios.

We study then rings’ shapes and anisotropies by looking at the ratios: (i) ⟨λ_1_^2^⟩/⟨λ_2_^2^⟩, (ii) ⟨λ_1_^2^⟩/⟨λ_3_^2^⟩ and (iii) ⟨λ_2_^2^⟩/⟨λ_3_^2^⟩. The first ratio indicates the elongation or “asphericity” of the ring mean shape, while the other two measure the extent to which rings become effectively flat due to slit confinement. Results are shown in Figure 3, where it is clear that for mild confinement (*Ĥ* ≳ 1.5) rings attain the same shape as the bulk ones (dashed lines). Conversely, for higher confinement (*Ĥ* ≲ 0.7), the ratios to the smallest eigenvalues (blue and red curves in Figure 3) are described by the same characteristic power-law behavior ∼*Ĥ*^–α^ with α = 2 (dotted lines). This exponent can be derived^23^ by the following simple blob scaling argument *à la* de Gennes:^24,25^ for tight confinement and since rings obey ideal statistics^13,14^ owing to strand crossings, we do expect ⟨λ_3_^2^⟩ ∼ *H*^2^ ∼ *a*^2^*g*_H_ while ⟨λ_1_^2^⟩ ∼ ⟨λ_2_^2^⟩ ∼ *H*^2^(*N*/*g*_H_) where *g*_H_ is the mean number of monomers spanning a distance of the order of *H*. Together these two relations imply α = 2, i.e., rings’ flattening is indeed compatible with the scaling picture. At the same time (ratios ⟨λ_1_^2^⟩/⟨λ_2_^2^⟩, green curve), the polymers maintain an elongated shape.

#### 3.1.2 Bond-Vector Correlation Function

**Figure 4:**
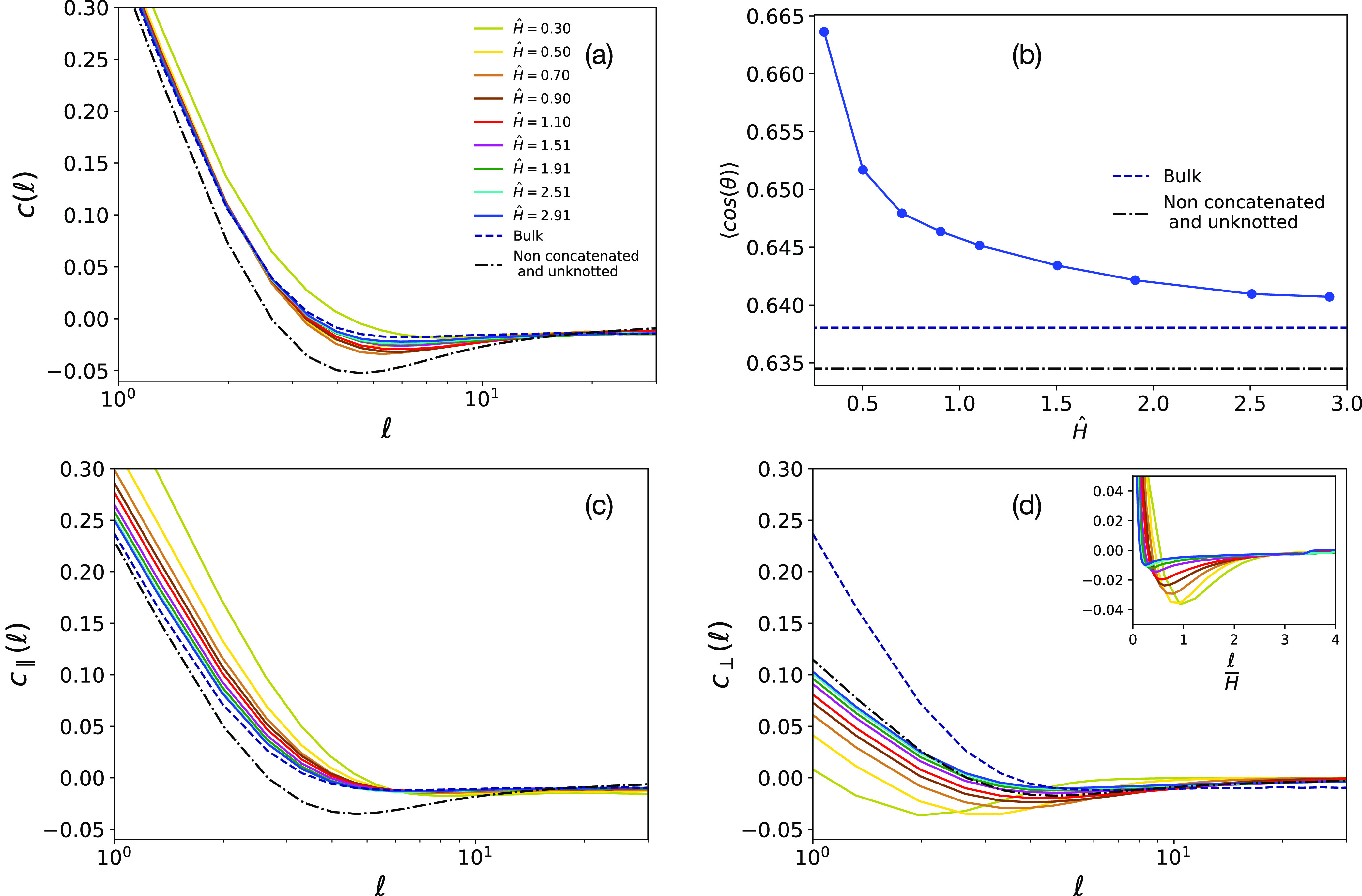
(a) , bond-vector correlation function as a function of the contour length distance . Colors are for different confinements, dashed and dot-dashed lines are for bulk melts and melts of nonconcatenated and unknotted rings (see legend). (b) ⟨cos(θ)⟩, mean cosine value between two consecutive bonds along the chain as a function of the degree of confinement *Ĥ*. (c) , contribution to the bond-vector correlation function in the *xy*-plane parallel to the slit. (d) , contribution to the bond-vector correlation function orthogonal to the plane of the slit; in the inset, the same quantity is represented as a function of the ring contour length normalized by the slit thickness, . Colors and symbols in (c) and (d) are as in (a).

We investigate now in more detail how the folding of polymer chains is affected by confinement by looking at the bond-vector correlation function
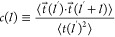
5as a function of the polymer contour length . This quantity gives useful insight when applied to bulk 3*d* melts of unknotted and nonconcatenated rings, in particular its distinct^16^ anticorrelation is a symptom of the double folding of the polymer chains at the entanglement scale (dot-dashed line in Figure 4a). In contrast (dashed line in Figure 4a), bulk 3d melts of randomly knotted and concatenated rings exhibit normal exponential decay behavior^14^ and are not characterized by double folding, hence the anticorrelation is absent.

To investigate the impact of confinement on chain folding, we have computed  for the confined rings. Results (Figure 4) exhibit several noteworthy effects. First (Figure 4a), for confined rings at small ,  decays more slowly than the bulk counterpart. This is the consequence (Figure 4b) of the increase of the mean cosine of the angle between consecutive bond vectors, ⟨cos(θ)⟩, as confinement increases: in other words, confined rings are slightly stiffer than the bulk reference, and this confinement-enhanced stiffness grows with the confinement. At the same time,  develops a characteristic anticorrelation that exhibits nonmonotonic dependence on *Ĥ*: in particular the deepest minimum occurs at *Ĥ* ≃ 0.7, i.e., the same value at which the gyration radius (Figure 2a) attains its minimum value. Moreover, the minimum itself disappears at the highest level of confinement. This peculiar behavior can be explained by considering the individual contributions of the parallel and transverse components of .  does not exhibit any minima (Figure 4c), while  displays a minimum for all values of *Ĥ* (Figure 4d). The mismatch in the values of , at which  is minimum while , causes the nonmonotonicity of the full . The latter goes to zero for similar values of  for all *Ĥ*, demonstrating that correlations grow mildly with the confinement. In contrast,  shows a minimum for  close to the thickness of the slit *H* (Figure 4d, inset). This is due to the back-folding of the polymer filaments induced by the hitting with the impenetrable walls of the slit: of course, this effect is more pronounced under strong confinement conditions, i.e., for . Thus, the minima in  appear when *H* has a similar value to the correlation length of , indicating the competition between these two length scales.

#### 3.1.3 Contact Probability

As just shown above, confinement alters the metric properties of the polymers. Then, it is natural to expect that the consequent reorganization of the chains modifies the intrachain polymer interactions. To test this hypothesis, we compute the mean contact probability between two monomers at contour length separation 
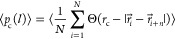
6where Θ(*x*) is the Heaviside step function and the “contact distance” *r*_c_ is set to the unit lattice size *a* (notice also that periodic conditions due to the ring geometry are tacitly assumed in eq 6).

**Figure 5:**
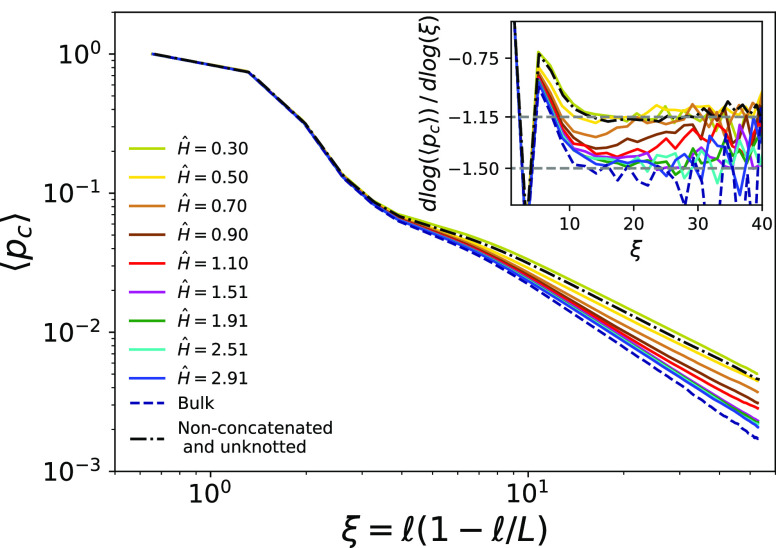
Mean contact probabilities, ⟨*p*_c_⟩ (eq 6), as a function of , where  is the contour length separation between monomers and *L* is the ring total contour length. Colors are for different confinements, dashed and dot-dashed lines are for bulk melts and melts of nonconcatenated and unknotted rings (see legend). Inset: local differential exponent .

Results are shown in Figure 5, where ⟨*p*_c_⟩ is plotted against the “effective” variable  in order to reduce^26^ finite size effect due to the ring geometry. First, one can notice that in bulk systems, as we let rings perform strand crossings, long-distance contacts decrease (dashed line) with respect to melts of nonconcatenated and unknotted rings (dot-dashed line). In contrast, confinement leads to an increase in the tail of the mean contact probability compared to that of the bulk reference. Notably, at *Ĥ* = 0.30, the tail’s slope is slightly less steep than in the nonconcatenated state.

To gain more insight, it is interesting to look at the exponent controlling the asymptotic power-law decay, ⟨*p*_c_⟩ ≃ ξ^–γ^ (Figure 5, inset). In bulk, strand-crossing rings attain ideal statistics characterized by γ ≃1.5, as confirmed by our previous findings.^13^ In contrast, confinement leads to a decrease in γ which becomes close to the same asymptotic value as the nonconcatenated state, γ ≃1.15. Based on mean-field arguments,^27^ γ = *dν*, where *d* is the space dimension and ν is the metric exponent of the chain relating^7,8^ the chain mean linear size to the number of monomers (i.e., ⟨*R*_g_^2^⟩ ∼ *N*^2ν^). Strand-crossing rings in bulk exhibit ideal statistics with ν = 1/2,^13^ and they are characterized by  in three dimensions. In confined systems, however, the rings cannot fold freely in three dimensions, effectively reducing the dimensionality of the system and resulting in a decrease in γ.

#### 3.1.4 Knots Statistics

In our kMC algorithm, two filaments from the same chain can cross, and this event may induce the formation of a knot along the chain. Characterization of knots spectra in confined systems has been addressed so far mostly for isolated chains,^23,28,29^ while fewer results are available for confined systems at melt conditions.

**Figure 6:**
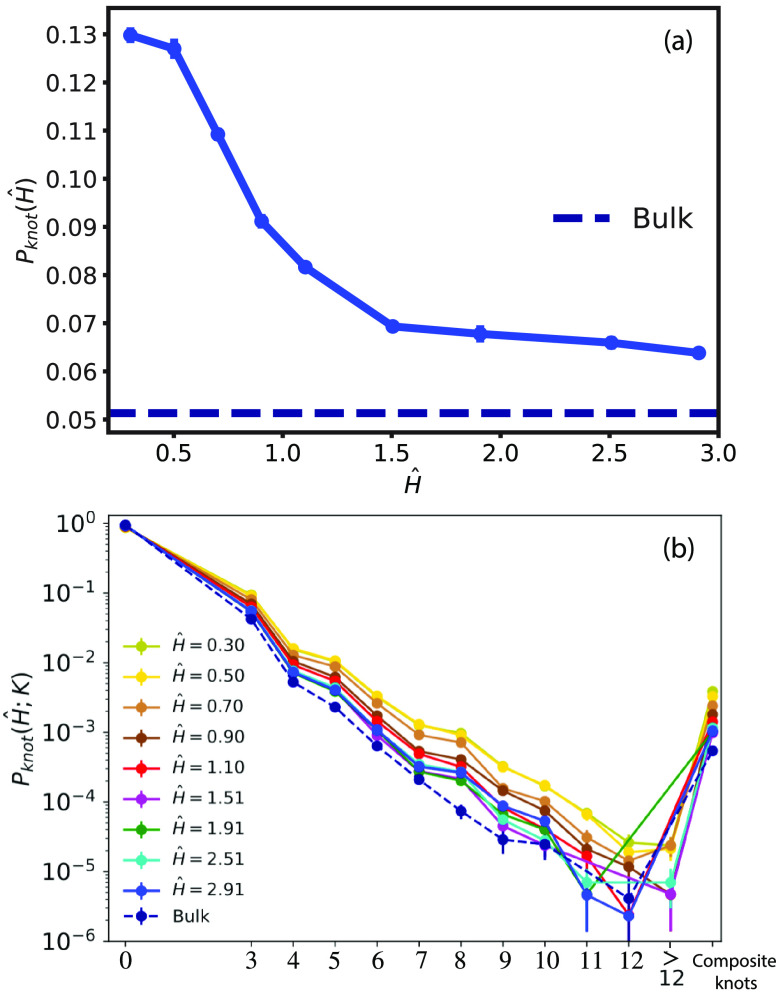
(a) *P*_knot_(*Ĥ*), ring knotting probability (eq 7) as a function of the degree of confinement *Ĥ*. The dashed line corresponds to the value for the bulk melt. (b) *P*_knot_(*Ĥ*; *K*), probability of finding a knot with crossing number *K*. Colors are for different confinements, and the dashed line is for bulk melts (see legend). *K* = 0 correspond to the unknot and *P*_knot_(*Ĥ*; *K* = 0) = 1 – *P*_knot_(*Ĥ*) is its corresponding probability. Knots with >12 crossings cannot be distinguished by *Topoly*.^20^ Composite knots are knots made up of 2 or more irreducible knots. Here, as well as in Figures S2 and S5 in the SI, error bars have been estimated by assuming the formula for simple binomial statistics for the probability of observing a given knot (link, in Figure S5 in the SI) type in the total population.

To fill this gap, we have investigated the occurrence of knots by computing the Jones polynomial of each ring of our systems, and for simplicity, we present our results based on the number of irreducible crossings (denoted by *K*, see Section 2.3). Specifically, we have computed the probability, *P*_knot_(*Ĥ*; *K*), of finding a knot with *K* irreducible crossings at given confinement degree *Ĥ* and the *cumulative* knotting probability
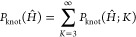
7which gives the probability that a ring in the melt contains a knot (of any type). As shown in Figure 6a, *P*_knot_(*Ĥ*) grows with the confinement and reaches the maximum value of ≃0.13 for the smallest *Ĥ*, resulting in an increase of ≃130% compared to bulk reference (dashed line). Both in bulk and in confinement, the most common knot type is the simplest one, namely, the *trefoil* knot 3_1_. Overall (Figure 6b), more complex knots are much less probable for all *Ĥ* values, yet their abundance increases with confinement, see Figure 6b for *P*_knot_(*Ĥ*; *K*) and Figure S2 in the SI for the relative population of knot types with *K* crossings. In conclusion, our analysis points out that confinement enhances the probability of knot formation, yet the overall occurrence of knots (i.e., *P*_knot_) remains relatively low (≲0.13).

### 3.2 Chain-Chain Correlations

#### 3.2.1 Chain Neighbors

The increase of the long-range intrachain contacts seen in Figure 5 may be indicative of the fact that confinement reduces the overlap between distinct chains or, in other words, ring–ring contacts should decrease. To test this hypothesis, we introduce the variable for the number of neighbors of ring *i* (*i* = 1, 2, ···, *M*)
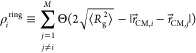
8where Θ(*x*) is the Heaviside step function, ⟨*R*_g_^2^⟩ is the ring mean-square gyration radius (eq 4), and *r⃗*_CM,*j*_ represents the center of mass position of the *j*th ring. According to eq 8, two rings are defined as “neighbor” whenever the spatial distance between their centers of mass is smaller than twice the root-mean-square gyration radius of the system. We have measured the distribution function of ρ^ring^, *P*(ρ^ring^), and its mean value, ⟨ρ^ring^⟩, at different confinements, and we study these quantities in relation to the distribution of spatial distances between the centers of mass *d*_CM–CM_ for neighboring rings, *P*(*d*_CM–CM_|neighbors).

**Figure 7:**
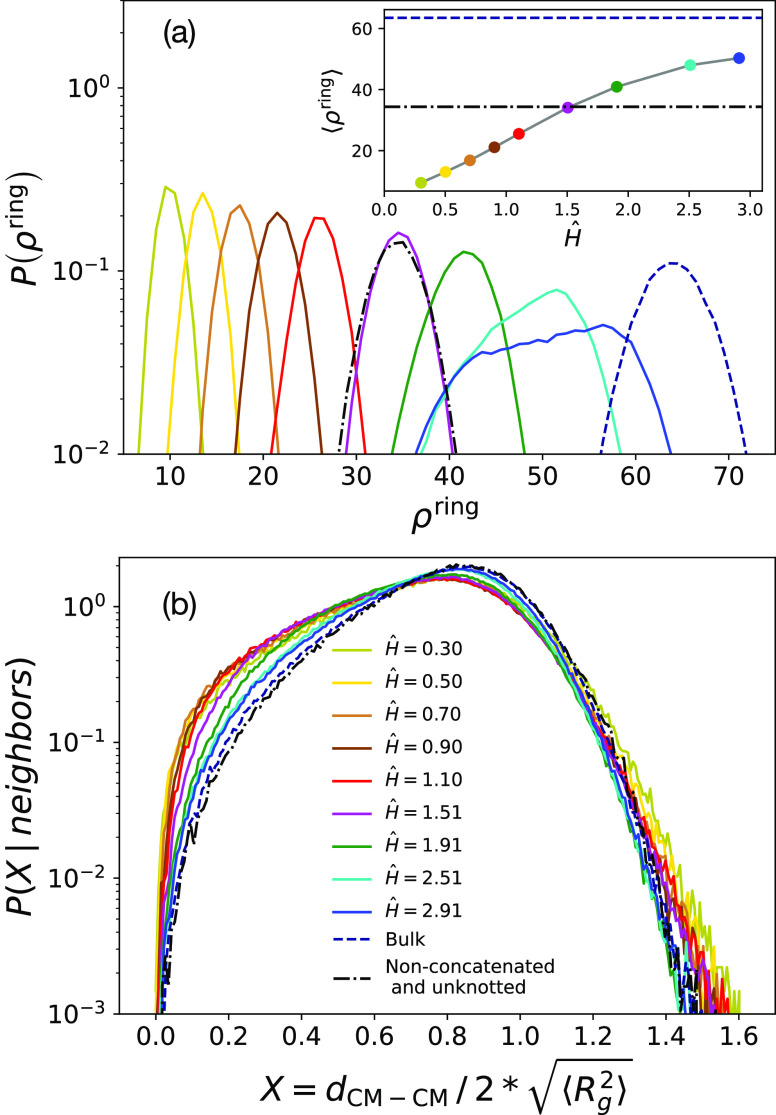
(a) Distribution function, *P*(ρ^ring^), of the number of neighbors per chain ρ^ring^. Inset: mean number of neighbors per ring, ⟨ρ^ring^⟩. (b) Distribution function of the distances between the centers of mass of neighboring chains, *P*(*d*_CM–CM_|neighbors), as a function of the variable normalized to twice the root-mean-square gyration radius,  (eq 4), of the rings. Colors are for different confinements, dashed and dot-dashed lines are for bulk melts and melts of nonconcatenated and unknotted rings (see legend).

Results are shown in Figure 7a, from which it is evident that ⟨ρ^ring^⟩ decreases as confinement increases, with ⟨ρ^ring^⟩ being always smaller with respect to the bulk reference (dashed line) and even smaller (for the tighter confinements *Ĥ* ≲ 1.5) with respect to the nonconcatenated and unknotted case (dot-dashed line). At the same time (Figure 7b), the distributions of spatial distances *d*_CM–CM_ demonstrate that neighboring chains tend to overlap more with each other under stronger confinement. Taken together, we can motivate the reason why the interchain contacts decrease in terms of the geometry of the slit. First, confinement can prevent the formation of stacked conformations along the transverse direction (see Figure S3 in the SI), and this surely reduces the interchain contacts. Moreover, we observe that, by reducing the width of the slit, inter-ring distances tend to increase, and this is an effect due to the increasing asymmetry of the slit as confinement increases (see Figure S4 in the SI).

#### 3.2.2 Links

The reduction of interchain contacts should also have consequences on the *linking* properties of the confined systems. To explore this aspect, we adopt the approach developed by us in ref (14) and compute: (a) ⟨*n*_2link_(|GLN|)⟩, the mean number of two-chain links per ring with absolute Gauss linking number |GLN| and (b) ⟨*n*_3link_⟩, the mean number of distinct three-chain links per ring with given chain topology.

**Figure 8:**
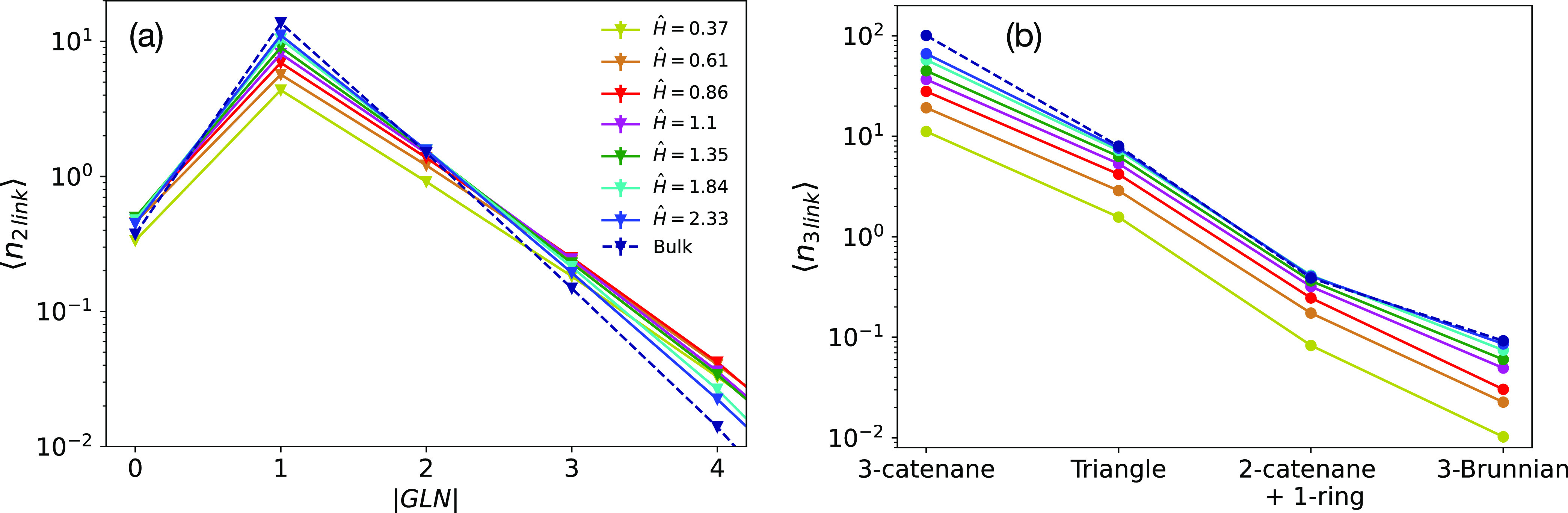
(a) ⟨*n*_2link_(|GLN|)⟩, mean number of links per ring with absolute Gauss linking number |GLN|. (b) ⟨*n*_3*l*ink_⟩, mean number of different three-chain linked structures per ring. Different colors are for the different confinements, and the dashed line is for the bulk system.

Results for ⟨*n*_2link_(|GLN|)⟩ are summarized in Figure 8a. We notice that ring–ring links are mostly Hopf-like (i.e., with |GLN| = 1) and that confinement reduces the extent to which the rings are linked, in agreement with the reported trend of neighbors per ring (Figure 7a). In general, the appearance of more complex links decreases exponentially, but the rate of decay depends on the level of confinement in the system. Chains under stronger confinement are characterized by a slower decay, which can be attributed to the fact that neighboring chains penetrate each other more (see Figure 7b). Additionally, links with |GLN| = 0 (i.e., the so-called Whitehead links) have been found between those with |GLN| = 2 and 3 at all confinements. We further classify these links by computing their Jones polynomial and determining their relative abundances (panel (a) in Figure S5 in the SI). We found that, even in this case, rings under stronger confinement form more complex links with greater ease.

To examine three-chain links, it is necessary to distinguish between two distinct groups of links: those that can be reduced to two-chain links and irreducible ones.^14^ The first group include: (a) *poly(3)catenanes*, chains made of three rings in which two nonconcatenated rings are connected to a common ring, and (b) *triangles*, triplets of rings which are all pairwise concatenated. Both (a) and (b) can be detected via pairwise linking. Instead, irreducible three-chain links cannot be detected via pairwise linking and can be further divided into two subtypes: (c) *poly(2)catenane+1-ring*, structures made of a poly(2)catenane plus another ring which is not directly concatenated (in a pairwise manner) to any of the other two, and (d) *Brunnian* links, nontrivial links which become a set of trivial links whenever one component ring is unlinked from the others (the so-called *Borromean* conformation, the link 6_2_^3^, constitutes the easiest example of this kind). By resorting to the shrinking method described in ref (14), we have detected links belonging to the last two classes and computed ⟨*n*_3link_⟩ for the different types of three-chain links (Figure 8b). It is clear from ⟨*n*_3link_⟩ that links organize onto a network made almost entirely via pairwise concatenation both in the bulk and in confinement. Irreducible three-chain links are much more rare and decrease with the degree of confinement; for this reason, the next analysis relative to polymer networks and entanglements (Section 3.2.3) has been performed by neglecting these three-chain links contributions. A detailed topological classification of these structures has been reported in Figure S5b in the SI, and even in this case, three-chain links with higher crossings seem to be more likely for more confined systems.

#### 3.2.3 Polymer Network and Entanglements

Concatenated rings give rise to a fully connected polymer network.^14,30^ To characterize this network, we define^14^ the linking degree LD_*i*_ of ring *i*
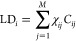
9where the sum runs over the total number of chains in the melt, and where *C*_*ij*_ is the *M* × *M* matrix expressing the concatenation status between rings *i* and *j*
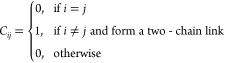
10The “weight” factor χ_*ij*_ takes into account the “complexity” of two-chain links: χ_*ij*_ = |GLN| or  depending on whether GLN ≠ 0 or GLN = 0, respectively. Here, *K* is the number of crossings characterizing the link, or in other words, each crossing of the link contributes 1/2 to an entanglement point. This quantity is of special interest as we have recently shown^14^ that the mean value  is directly connected to the entanglement length of the melt, *N*_e_, via the relation ⟨LD⟩ = *N*/*N*_e_. To complement this analysis, we have also computed the distribution of the values LD at the single-ring level, *P*(LD), which gives us information about the heterogeneity of the network.

**Figure 9:**
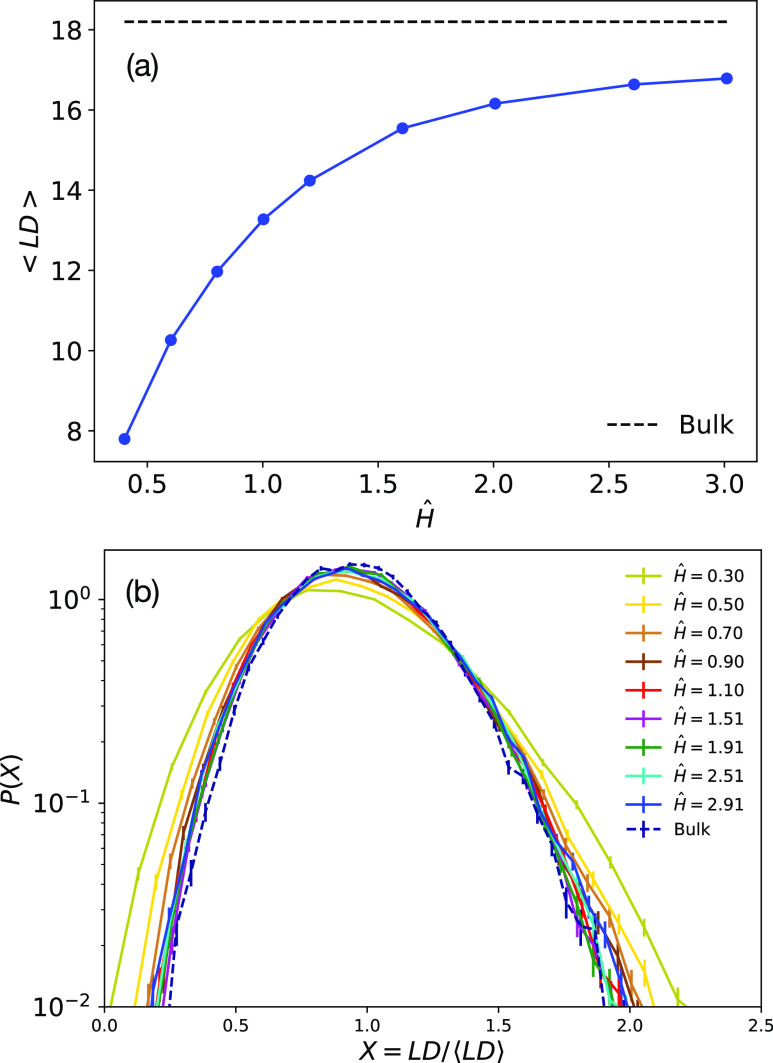
(a) Mean linking degree, ⟨LD⟩, as a function of the confinement. The horizontal dotted line represents the bulk value. (b) Distribution functions, *P*(LD), of the linking degree as a function of the variable normalized to the corresponding mean value ⟨LD⟩. Different colors are for the different confinements; the dashed line is for the bulk system.

Results are listed in Figure 9. ⟨LD⟩ (panel (a)) decreases as a function of the confinement, up to a reduction of ≃60% with respect to bulk conditions. Then, by looking at the distribution functions (panel (b)) of the linking degree as a function of X = LD/⟨LD⟩, we see that the curves at mild confinements display the same behavior for bulk conditions. Conversely, the tails become stronger for more confined systems. This is in agreement with the behavior seen for the distribution functions of the ring size (Figure 2b), where the tails are higher for stronger confinements. Fluctuations of ring size may impact concatenation since smaller rings will be less concatenated, having less possibility to reach other rings, while bigger rings can host more contacts and consequently more concatenations. To sum up, the resulting networks of concatenated rings tend to be more heterogeneous as the confinement becomes stronger, in line with the fluctuations of ring size.

## 4 Discussion and Conclusions

Our findings illustrate the impact that slit confinement has on the spatial structure of randomly concatenated and knotted ring polymers under melt conditions.

At the single-chain level, our investigation shows that as rings flatten with increasing confinement, they tend to adopt more elongated conformations. At the same time, rings become slightly more rigid with the confinement, a tendency captured by the increase of the correlation (⟨cos(θ)⟩, Figure 4b) between consecutive bonds along the chain. We have also demonstrated that the competition between the Kuhn length of the polymers, , and the height of the slight, H, induces a nonmonotonous behavior on the bond-vector correlation function,  (see Figure 4a,c,d). In general, the impact of confinement on ring conformations becomes particularly pronounced with respect to the formation of long intrachain contacts as the slit narrows (see Figure 5), resulting in more compact rings. Finally, these changes have significant repercussions on the knotting probability which increases with the confinement, and for which we register an increase of ≃130% compared to the bulk value (see Figure 6a).

The effects of slit confinement on the interchain statistics are similarly noteworthy. Specifically, as the level of confinement increases, the average number of neighbors per ring, ⟨ρ^ring^⟩, experiences a considerable decrease (see Figure 7a). This is directly connected to the decrease of the mean linking degree, ⟨LD⟩, which displays a total reduction of ≃60% with respect to bulk conditions.

This finding has relevant implications. Being ⟨LD⟩ directly related to the mean number of entanglement strands per ring,^14^ its decrease as confinement grows means that, at fixed monomer density, confinement alone may alter the entanglement properties of the system making *N*_e_ effectively bigger. This would explain recent findings^31,32^ showing that for both linear chains and rings in two-dimensional melts, the resulting dynamical quantities display a quite surprising Rouse-like behavior^7,8^ which, ultimately, points toward the effective irrelevance of entanglement effects due to interchain interactions. Along the same lines, it is worth recalling that the elastic plateau modulus *G*_0_, which quantifies the stress–strain relationship of polymeric materials, is related^8^ to the total number of entanglement strands of the melt, . In other words, our results imply that, as confinement grows, the resulting polymer network becomes softer (*G*_0_ decreases), revealing a fundamental connection existing between geometric confinement, topology, and the mechanical properties of the stored network. Interestingly, this connection appears to be not limited to only polymer melts but it seems to be a quite general feature appearing in other notable classes of soft materials like, e.g., DNA nanostar hydrogels.^33^

We conclude by proposing a possible experimental realization of the systems studied in this work. As discussed in the Introduction, a first experiment^15^ on slit-confined kinetoplast DNA—a naturally occurring catenated network of DNA rings—at different degrees of confinement has already been performed. However, there the topology of the kinetoplast was maintained fixed since the study was focusing on the shape and size rearrangements of the network once placed under confinement. Our predictions here could be tested in a relatively simple variant of this experiment in the following way: as in Krajina et al.,^3^ it would be sufficient to introduce suitable amounts of the enzyme topoisomerase-II and, by doing so, promoting cut-and-resealing events in the system that would reshape the DNA network topology. Then, again as described in ref (3), by probing the system through microrheology, it should be possible to measure the mechanical properties of the catenated network and verify the predicted softening under confinement.

## Supporting Information Available

Time mean-square displacement of monomers in the frame of the center of mass of the corresponding ring, fractional population of knot types, contour plots for the joint distribution function of parallel and transverse components of the distances between the centers of mass of neighboring rings, distribution functions of the distances between the rings’ centers of mass, and fractional population of two-chain links with GLN = 0 (PDF)
