# Supplementary material for: Spatial Organization of Slit-Confined Melts of Ring Polymers with Nonconserved Topology: A Lattice Monte Carlo Study
Source: Macromolecules. 2023 Sep 28;56(19):7860–9. doi: 10.1021/acs.macromol.3c01320 (PMC10569094; doi:10.1021/acs.macromol.3c01320)
Supplement: Supplementary file 1 — ma3c01320_si_001.pdf [file ma3c01320_si_001.pdf]

# – Supporting Information –

Spatial organization of slit-confined melts of ring polymers with non-conserved topology: A lattice Monte Carlo study

Mattia Alberto Ubertini and Angelo Rosa*
Scuola Internazionale Superiore di Studi Avanzati (SISSA), Via Bonomea 265, 34136 Trieste, Italy

Table of Content

Fig. S1 .... page S2
Fig. S2 .... page S3
Fig. S3 .... page S4
Fig. S4 .... page S5
Fig. S5 .... page S6
References .... page S7

* anrosa@sissa.it

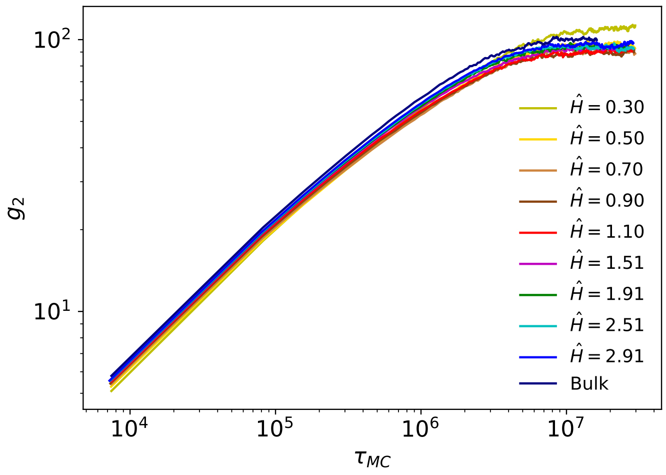

FIG. S1. Time mean-square displacement of monomers in the centre of mass of the corresponding ring [1, 2],

$$g_2(\tau_{\mathrm{MC}}) \equiv \left\langle \frac{1}{M} \sum_{m=1}^{M} \left[(\vec{r}_m(\tau_{\mathrm{MC}}) - \vec{r}_{\mathrm{CM}}(\tau_{\mathrm{MC}})) - (\vec{r}_m(0) - \vec{r}_{\mathrm{CM}}(0)) \right]^2 \right\rangle ,$$

as a function of the Monte Carlo (MC) time, τ_{MC}. $\vec{r}_m$ is the spatial position of monomer m, while $\vec{r}_{\mathrm{CM}}$ is the spatial position of the centre of mass of the ring. All examined systems display a plateau, that is indicative of their successful equilibration. The time scale associated to the onset of the plateau is used to estimate which amount from the initial portion of the corresponding MC trajectory has to be discarded in order to compute rings' properties accurately.

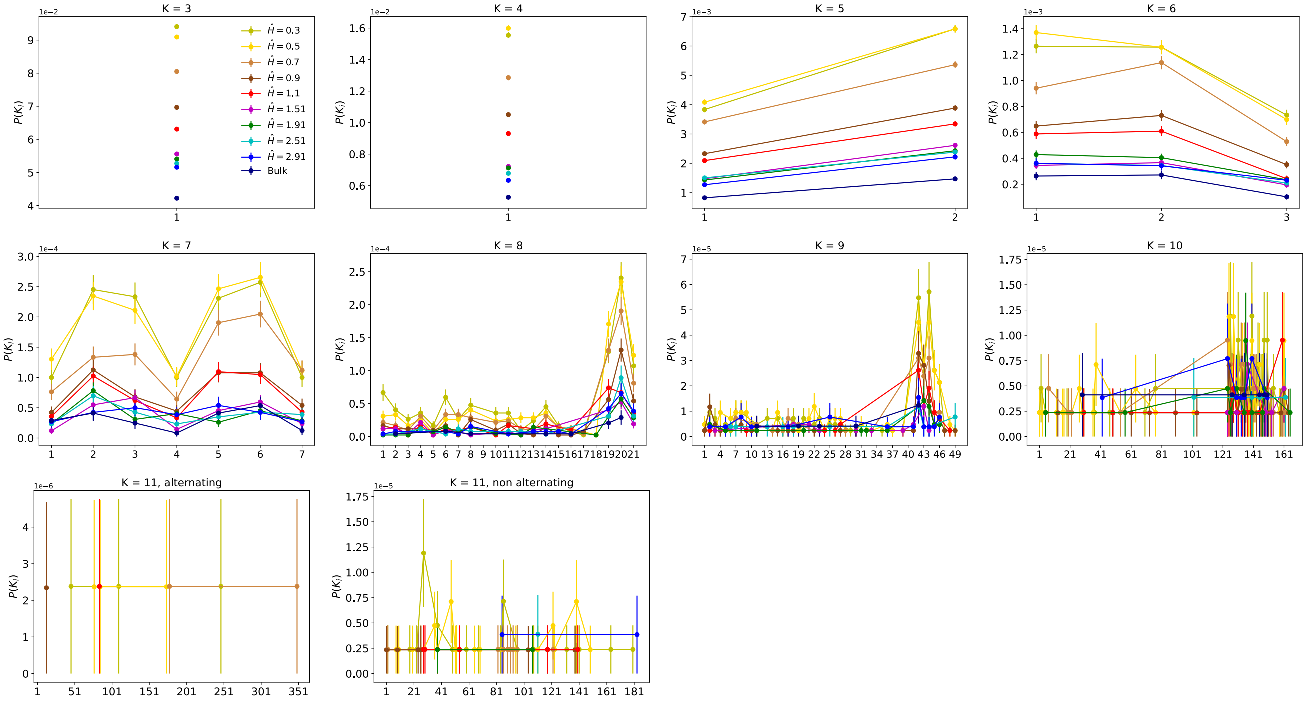

FIG. S2. Fractional population of each knot type – named according to the Rolfsen convention [3] – at fixed number of crossings K and for the different values of confinement $\hat{\mathrm{H}}$ and in bulk conditions (see legend). For $K = 11$, knots are categorized according to the Hoste-Thistlethwaite table [4] that split them in *alternating* (K_{a_i}) and *non-alternating* ones (K_{n_i}), with the index i used to enumerate the rings separately within each group. Large relative error bars are due to the limited size of the sample (notice that the values of each y-axis have to be multiplied by the power-law reported on the top left corner of the corresponding panel).

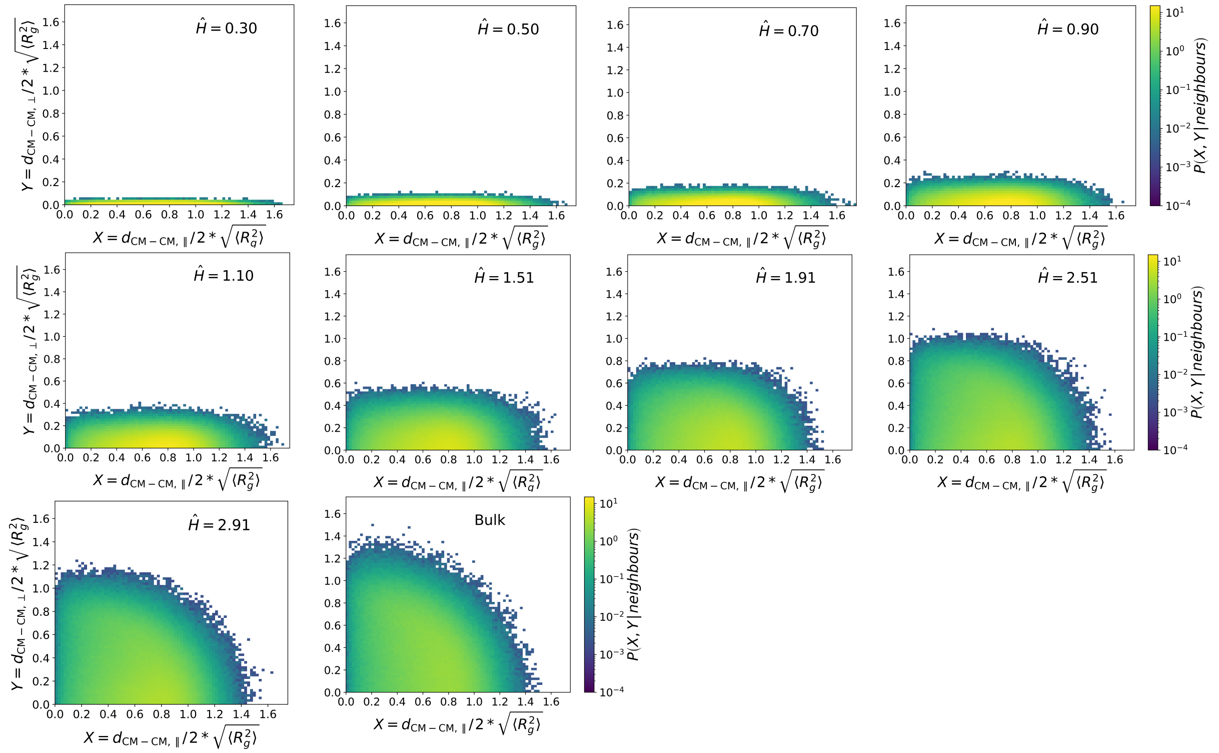

FIG. S3. Contour plots for the joint distribution function of the slit-parallel and slit-transverse (or, slit-perpendicular) components of the distances between the centres of mass of neighboring rings $P(d_{\mathrm{CM-CM},\parallel}, d_{\mathrm{CM-CM},\perp} \,|\, \mathrm{neighbors})$. Distances have been rescaled by the corresponding root-mean-square gyration radius of the rings.

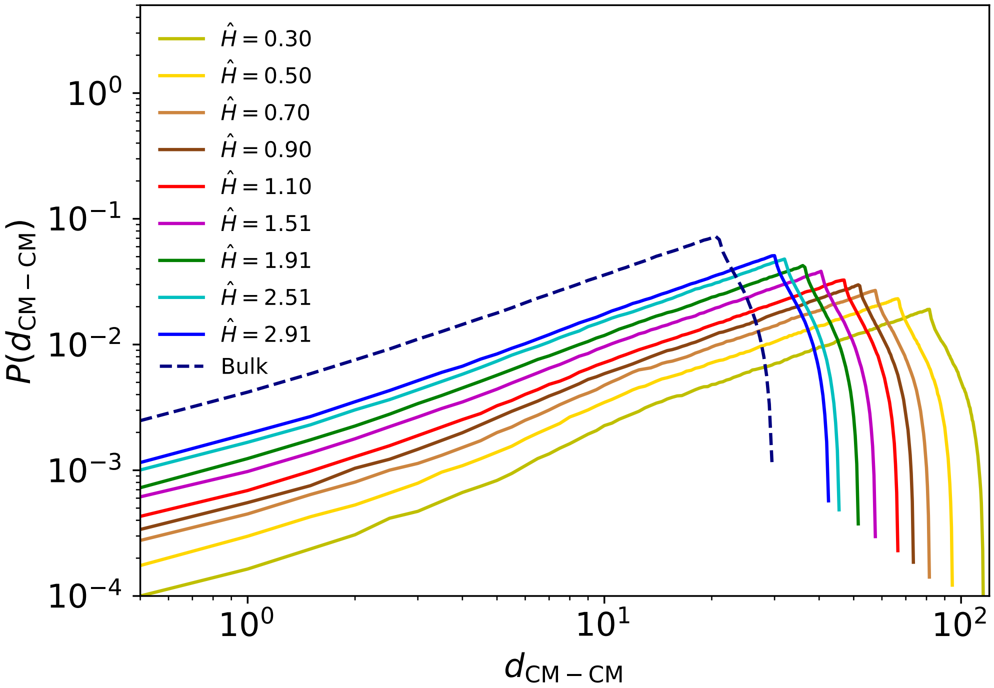

FIG. S4. Distribution functions of the distances between the rings' centres of mass, $P(d_{\mathrm{CM-CM}})$.

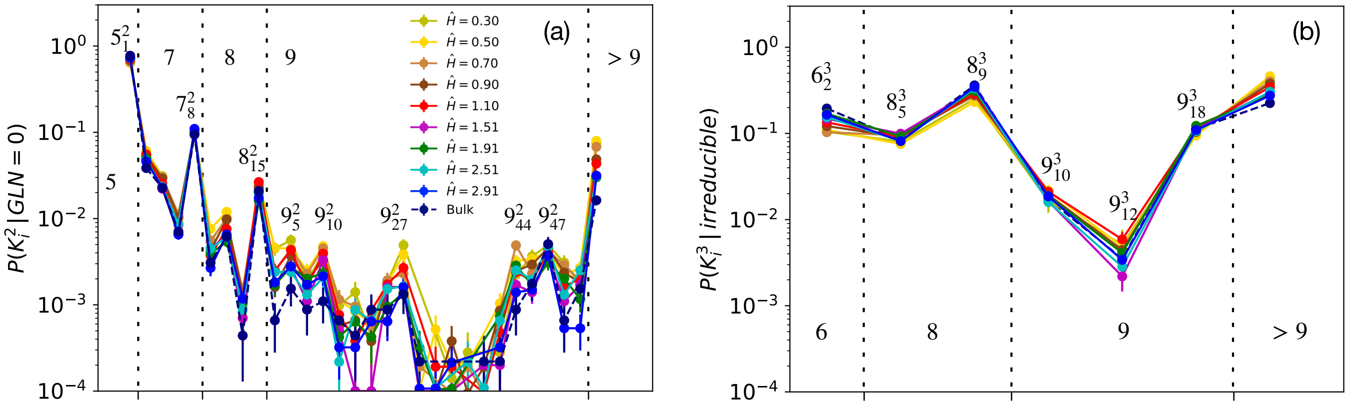

FIG. S5. (a) $P(K_i^2|\mathrm{GLN}=0)$, fractional population of two-chain links K_i^2 (termed according to the Rolfsen convention [3]) having GLN = 0. Labels have been put for links corresponding to peaks of the distributions. (b) $P(K_i^3|\mathrm{irreducible})$, fractional population of three-chain links K_i^3 (also termed according to the Rolfsen convention [3]) belonging to the poly(2)catenane+1-ring and Brunnian classes (see main text for details). *Topoly* fails [5] recognizing links with >9 crossings, so these lack categorization. In both panels, vertical dotted lines delimit areas of links at fixed number of crossings K.

[1] K. Kremer and G. S. Grest, The Journal of Chemical Physics **92**, 5057 (1990).

[2] M. A. Ubertini and A. Rosa, Phys. Rev. E **104**, 054503 (2021).

[3] D. Rolfsen, *Knots and links* (AMS Chelsea Publishing, 2003).

[4] J. Hoste, M. Thistlethwaite, and J. Weeks, Math. Intelligencer **20**, 33 (1998).

[5] P. Dabrowski-Tumanski, P. Rubach, W. Niemyska, B. A. Gren, and J. I. Sulkowska, Briefings in Bioinformatics **22**, bbaa196 (2021).
